# Research on Tomato Quality Prediction Models Based on the Coupling of Environmental Factors and Appearance Phenotypes

**DOI:** 10.3390/plants14233569

**Published:** 2025-11-22

**Authors:** Longwei Liang, Zhaoyuan Wang, Kaige Liu, Jing Xu, Changhong Li, Huiying Liu, Ming Diao

**Affiliations:** 1 College of Agriculture, Shihezi University, Shihezi 832003, China; lianglongwei865@gmail.com (L.L.); maogaocai@foxmail.com (Z.W.); liukaige910@126.com (K.L.); 20212012035@stu.shzu.edu.cn (J.X.); 18706986080@163.com (C.L.); 2 Research Center of Information Technology, Beijing Academy of Agriculture and Forestry Sciences, Beijing 102206, China

**Keywords:** tomato quality prediction, environmental factors, appearance phenotypes, long short-term memory, gated recurrent unit, deep neural network

## Abstract

This study addresses the limitations of current non-destructive techniques for assessing tomato quality, such as their high cost, strong dependence on spectroscopic instruments, and difficulty in dynamic monitoring. The study proposes an integrated tomato quality prediction model that combines a Long Short-Term Memory (LSTM)-based environmental predictor, a Gated Recurrent Unit with attention mechanism (GRU-AT) for dynamic maturity prediction, and a Deep Neural Network (DNN)-based quality evaluation module. The LSTM model demonstrated high accuracy in environmental prediction (R^2^ > 0.9559). The GRU-AT model excelled in color ratio prediction (R^2^ > 0.86), and the DNN model achieved R^2^ values exceeding 0.811 for lycopene (LYC), firmness (FI), and soluble solids content (SSC). Experimental results demonstrate that this approach can accurately predict multiple quality parameters using only standard RGB images. In summary, this study provides a low-cost, low-complexity solution enabling real-time, non-destructive monitoring of greenhouse tomato quality, offering a viable pathway for crop quality management in precision agriculture.

## 1. Introduction

Tomatoes (*Solanum lycopersicum* L.) are one of the most important crops grown in greenhouses around the world. Their quality formation is a complex physiological process influenced by genetic regulation and environmental factors. Studies suggest that tomato quality hinges on critical physiological indicators, including firmness index (FI), soluble solids content (SSC), soluble sugars (SS), titratable acidity (TA), vitamin C (VC), and lycopene (LYC) [1,2]. However, although precise, traditional detection methods such as high-performance liquid chromatography (HPLC) and spectrophotometry (SP) are destructive, time-consuming, and difficult to implement for high-throughput analysis [3,4]. Thus, they cannot meet the real-time monitoring and precise regulation requirements of facility agriculture.

In recent years, non-destructive testing technologies, such as near-infrared spectroscopy (NIRS), have been widely used to assess fruit quality [5,6]. For instance, Chen employed NIR combined with backpropagation neural networks and partial least squares (BP-PLS) algorithms to evaluate the soluble solids content of blueberries [7], and Vega Castellote utilized NIR technology to grade the internal quality and maturity of whole watermelons online at an industrial scale [8]. However, these methods rely on expensive optical equipment and typically only provide static measurements. This makes it difficult to continuously capture the physiological processes of fruits as they evolve in response to light and temperature conditions in greenhouses. This severely limits the large-scale integration and application of this technology in facility-based agricultural production systems.

Surface color is the most intuitive phenotypic characteristic of tomato ripeness, and its changes are significantly correlated with internal physiological metabolism [9,10]. Previous studies have confirmed that the transformation of fruit surface pigment composition (e.g., chlorophyll and carotenoids) is genetically regulated and cumulatively influenced by environmental factors such as light, temperature, and humidity [11,12]. For instance, Ghasemi Soloklui investigated how environmental factors affect the color, physical characteristics, and physicochemical components of pomegranates. They found that, compared to warmer environments, pomegranates grown in their native climate had higher TA, SSC, and pH values. Environmental factors such as wind speed, altitude, and annual precipitation were significantly correlated with SSC, fruit weight, aril weight, edible portion weight, pH, TA, phenolic content, antioxidant content, and anthocyanin content [13]. However, current research has two key limitations. First, most studies focus solely on correlating color and composition at a single time point or establishing static predictive models based on spectral reflectance. These studies fail to systematically analyze the spatiotemporal coupling of color dynamics with internal quality parameters [14]. Second, existing methods generally overlook the cumulative regulatory effects of environmental factors on tomato ripening [15]. Thus, current research has not yet achieved a quantitative analysis of the synergistic effects of environmental factors, dynamic color responses, and internal component transformations during the continuous ripening process of tomatoes. These limitations severely restrict the universality and timeliness of quality prediction models based on color characteristics.

To address these challenges, next-generation agricultural phenotyping technologies are rapidly evolving toward multimodal sensing and intelligent analysis. Deep learning models, particularly temporal neural networks such as GRU and LSTM, have unique advantages for monitoring crop growth due to their powerful feature extraction and temporal modeling capabilities. For instance, Qin et al. [16] proposed the SIS-YOLOv8 model, which outperformed the original YOLOv8n model in potato and tomato disease detection tasks. The SIS-YOLOv8 model achieved an 8.2% increase in accuracy, a 4% increase in recall, and a 5.9% increase in mAP50. This demonstrates its stronger adaptability to complex agricultural environments and provides an effective solution for automated crop disease detection. However, existing studies [17,18] have primarily focused on single-modal data (e.g., spectral or image data) and have failed to effectively integrate the interactions between multiscale phenotypic features (e.g., surface color proportion) and environmental covariates (e.g., cumulative light-temperature effects). Notably, color evolution during tomato maturation exhibits significant temporal characteristics. Early chlorophyll degradation and late carotenoid accumulation follow different patterns (non-stationarity) [19]. Dark-colored varieties may also exhibit overlapping chlorophyll retention and anthocyanin accumulation, a process closely related to chloroplast degradation [20]. This requires prediction models that possess dual capabilities: feature-adaptive selection and temporal dependency modeling.

To address these limitations, we developed a low-cost, high-efficiency dynamic monitoring method. This method should address the following two research requirements simultaneously: (1) analyze the multidimensional coupling relationships among environmental factors, phenotypic traits, and internal and external quality during tomato maturation and (2) establish a predictive model for internal and external quality based on dynamic changes in appearance characteristics. This systematic research framework will elucidate the regulatory networks that govern the formation of fruit quality under environmental stress and provide technical support for precise harvesting. This study proposes a novel, computer vision-driven method that enables the non-destructive monitoring of tomato quality throughout the entire maturation period via RGB image analysis. Specifically, the research will address the following key scientific issues: (1) establishing a model that relates cumulative environmental effects to fruit surface color and quantifies the regulatory patterns of external driving factors on maturity, (2) constructing nonlinear mapping relationships between multiscale color features and firmness index (FI), soluble solids content (SSC), Soluble sugars (SS), acidity (AT), vitamin C (VC), and lycopene (LYC) to reveal the synergistic evolutionary pathways between appearance phenotypes and internal and external quality, and (3) integrating a three-dimensional model of environment-phenotype-internal and external quality to predict internal and external quality parameters and the optimal harvest window for tomatoes at any growth stage using only sequential images. Compared to existing spectral technologies, this method only requires ordinary camera equipment. It extracts high-dimensional color features through machine learning algorithms and significantly reduces hardware costs and deployment complexity while markedly enhancing the model’s robustness and applicability in actual greenhouse production environments.

## 2. Materials and Methods

### 2.1. Overview of the Experimental Process for the Tomato Fruit Ripeness Prediction Model

**Figure 1:**
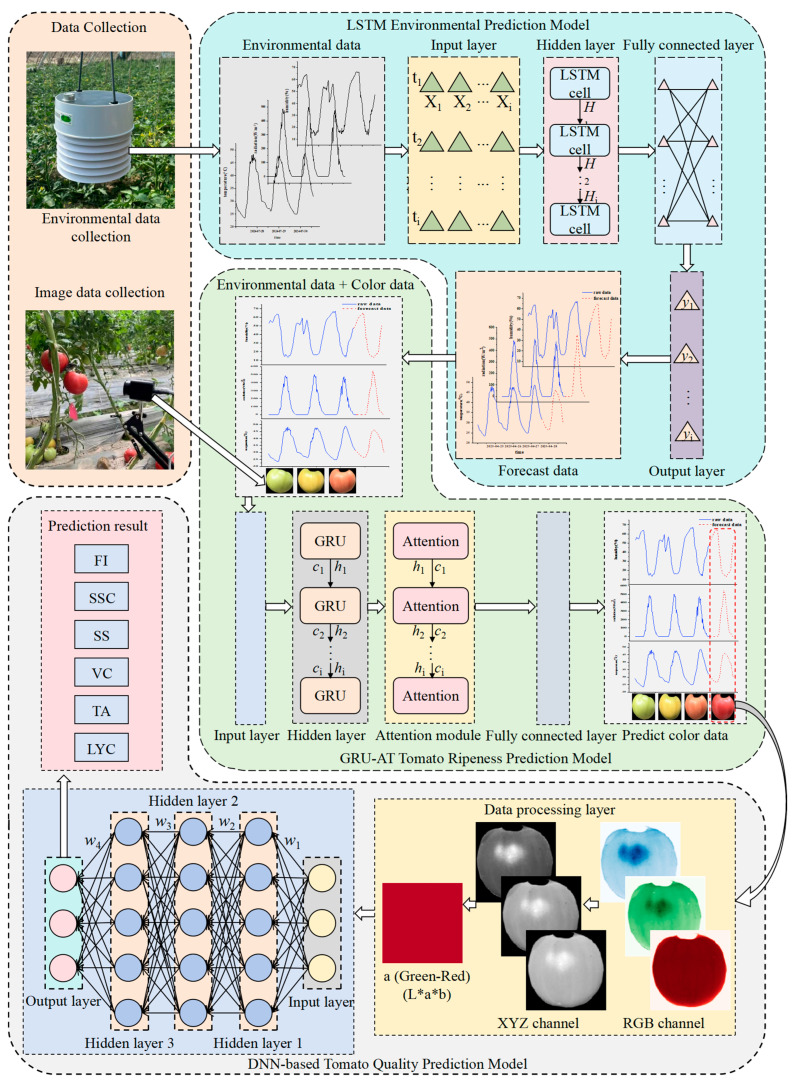
Technology Roadmap. Note: The framework outlines a sequential, data-driven prediction pipeline. The process begins with multi-source data collection, including environmental parameters and tomato images. The Environmental Prediction Model (LSTM) first forecasts future conditions from historical data to provide key temporal drivers. These predicted environmental data, together with captured tomato color data, are fed into the Tomato Ripeness Prediction Model (GRU-AT). This model employs Gated Recurrent Units to capture cumulative environmental effects and an attention mechanism to focus on critical time points, ultimately generating predicted color data. This output then serves as input to the DNN-based Quality Prediction Model, which decodes complex relationships between color information and internal quality metrics to produce the final comprehensive quality assessment.

Figure 1 shows the entire experimental process of the tomato quality prediction model system.

### 2.2. Test Overview

From March 2024 to May 2025, this experiment was conducted at the Kashgar (Shandong Shuifa) Vegetable Industry Demonstration Park (39.35°E, 76.02°N) in Xinjiang. Traditional solar greenhouses (east–west orientation, 50 m long × 8 m wide) were used for the experiment, which focused on Provence tomatoes. The experimental period was designed based on the key physiological stages of tomato growth and development. The focus was on monitoring the complete developmental process, from fruit enlargement to full maturity. Standardized cultivation management protocols were strictly followed throughout the experiment: a timed drip irrigation system was used (90 min daily), and periodic nutrient management was implemented (application of water-soluble fertilizers containing macronutrients every three days, with an N–P_2_O_5_–K_2_O ratio of 20%–20%–20% and an application concentration of five kilograms of fertilizer per 180 L of water). Standardized plant regulation measures were implemented during the fruit development stage. These measures included timely pruning and suckering, fruit thinning (retaining four to five fruits per cluster), and integrated pest and disease management. These measures ensured that the environmental parameters and agronomic practices of plant growth met the optimal requirements for tomato growth and development.

### 2.3. Data Collection

**Figure 2:**
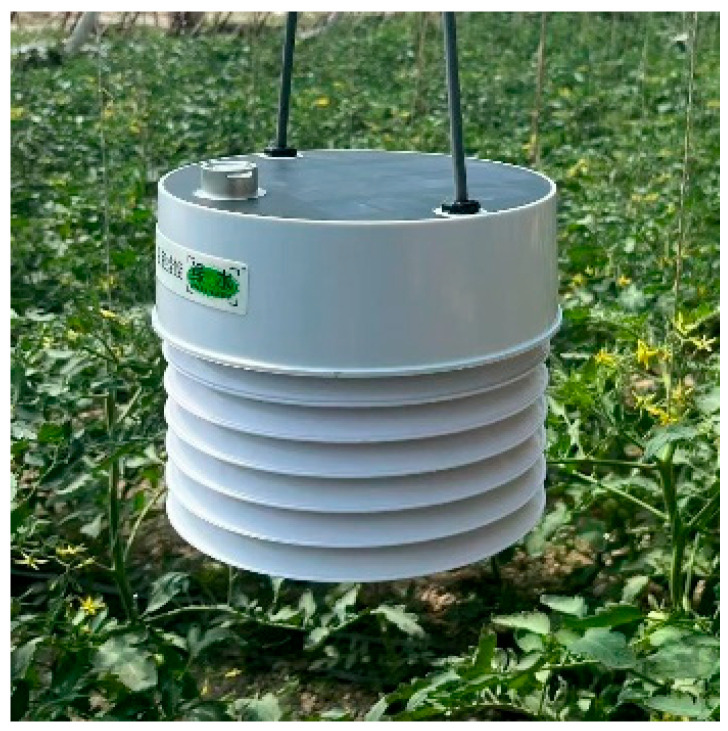
Data collector.

This test employed a multi-parameter greenhouse environment data logger from Nongxin Technology (Beijing) Co., Ltd. (Beijing, China) as the data acquisition system. The monitored parameters include temperature, humidity, and solar radiation intensity. Figure 2 shows the data logger, and Table 1 shows the detailed technical specifications of the sensors it carries.

**Figure 3:**
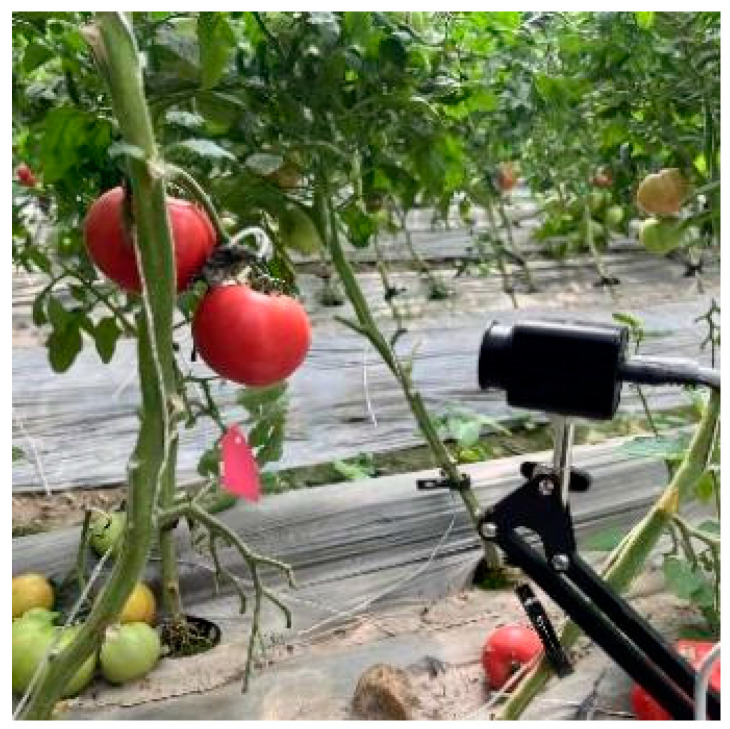
Tomato image acquisition.

In this experiment, the complete dynamic process from the mature green to the late red stage was continuously monitored for 80 tomato plants (with one fruit selected per plant) using a KSJLENS industrial camera. A total of 1606 images with a resolution of 3024 × 3024 pixels were captured (as depicted in Figure 3), and the corresponding data distribution is detailed in Table 2.

### 2.4. Data Preprocessing

To ensure data integrity and reliability, this study applied a systematic quality control process to address outliers and missing values from data collection and transmission. First, the box-plot method was used to identify outliers based on statistical principles, with the following criteria: the upper bound (Q_3_ + 1.5 × IQR) and the lower bound (Q_1_−1.5 × IQR), where Q_1_ and Q_3_ represent the 25th and 75th percentiles of the data, respectively, and IQR (interquartile range) is the difference between Q_3_ and Q_1_ [21]. Detected outliers are treated as missing values. Missing data are filled in using differentiated strategies based on the duration of the missing values. For data segments with no more than five consecutive missing points, linear interpolation is used. For segments with more than five consecutive missing points, historical data from the nearest timestamp with matching weather conditions is used (as described in Equation (1)). This strategy optimizes the interpolation method by distinguishing the length of missing data. It effectively suppresses errors introduced by filling in long-term missing data and significantly improves the continuity and reliability of the data series. This lays a high-quality data foundation for subsequent modeling and analysis.

(1) $$\begin{array}{c} y_{t}=\left\{\begin{array}{cc} y_{a}+\frac{\left(y_{b}-y_{a}\right)\times \left(t-t_{a}\right)}{t_{b}-t_{a}} & \left(t\leq 5\right)\\ y_{h} & \left(t>5\right) \end{array}\right. \end{array}$$

In the formula, *y_t_* is the missing value. *y_a_* and *t_a_* are the time and value of the first valid point before and after the missing segment. *y_b_* and *t_b_* are the time and value of the first valid point after the missing segment. *t* is the time to be interpolated. *y_h_* is the historical data of the nearest point with the same weather conditions.

Due to differences in data units and dimensions, the data were normalized using Equation (2), converting all values to a range between 0 and 1. To prevent data leakage and ensure the fairness of model evaluation, a strict data splitting strategy was adopted: First, based on the unique “plant ID,” all 80 plants were divided into training, validation, and test sets in an approximate ratio of 7:2:1, ensuring that the plants in the test set were entirely unseen during the training and validation phases. Simultaneously, a temporal gap was enforced during the split, such that the data in the test set overall originated from a later time period than those in the training and validation sets, thereby simulating the model’s predictive performance on future unknown data. Finally, the normalized data were assigned to the three datasets according to this splitting strategy.

(2) $$y=\frac{d-d_{min}}{d_{max}-d_{min}}$$

In this formula, d represents the original data. *d_min_* and *d_max_* represent the minimum and maximum values of the original data, respectively. *y* represents the normalized data.

**Figure 4:**
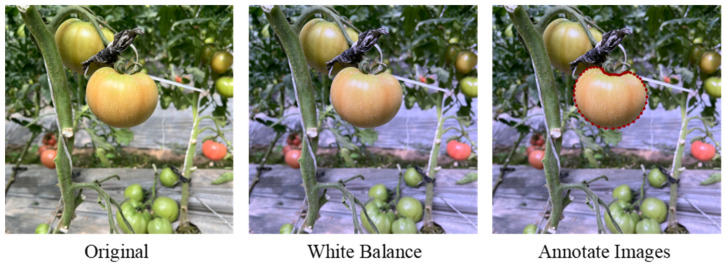
Tomato image preprocessing.

This study established a standardized annotation and color processing workflow for tomato fruit image data. During the image annotation phase, the LabelMe tool was used for pixel-level annotation of the tomato fruit outline. For color processing, color correction was applied to the tomato images to efficiently restore color through computer vision and image processing techniques. First, the original data was acquired and converted to a different color space. Then, an automatic white balance algorithm based on the grayscale world assumption was applied to correct the image colors. This involved calculating the statistical characteristics of each color channel and dynamically adjusting the channel gain coefficients to achieve accurate color restoration. This approach ensured the robustness of the algorithm while significantly improving the accuracy of color restoration, providing a high-quality visual data foundation for subsequent image analysis. Figure 4 demonstrates the method for image color correction, with the specific formulas presented in Equations (3)–(7):

(3) $$a_{gray}=\frac{a_{r}+a_{g}+a_{b}}{3}$$

(4) $$s_{r}=\frac{a_{gray}}{a_{r}}$$

(5) $$s_{g}=\frac{a_{gray}}{a_{g}}$$

(6) $$s_{b}=\frac{a_{gray}}{a_{b}}$$

(7) $$I_{balanced}=I_{original}\cdot s$$

In the formula, *a_gray_* is the average gray value of the three channels, *a_r_* is the average pixel value of the red channel, ag is the average pixel value of the green channel, ab is the average pixel value of the blue channel, *s* is the gain coefficient, *s_r_* is the gain coefficient of the red channel, *s_g_* is the gain coefficient of the green channel, *s_b_* is the gain coefficient of the blue channel, *I_balanced_* is the image after white balance, and *I_original_* is the original image.

### 2.5. Physical and Chemical Indicator Measurements

#### 2.5.1. Surface Color

The surface color of tomato fruits was objectively evaluated using a precision colorimeter (Model: NR110, Shenzhen Tianyouli Standard Light Source Co., Ltd., Shenzhen, China) based on the CIELAB (L*a*b*) color system, following the relevant standard method [22]. The instrument was configured with a D65 standard illuminant and a 10° standard observer angle, and calibrated before each measurement session using the provided white and black reference tiles. Fruits of uniform maturity and free from surface defects were selected and placed on the instrument’s sample stage against a neutral gray background. Two symmetrical points spaced 180° apart along the equatorial region of each fruit were measured using an 8 mm diameter measuring aperture, which was placed in full contact with the fruit skin to avoid shadowing effects. Two independent measurements were obtained per fruit, with the measuring head lifted and repositioned between readings. The final color values were calculated as the arithmetic mean of the two measurements. All measurements were conducted in a darkroom environment to eliminate interference from ambient light.

#### 2.5.2. Firmness (FI)

FI was measured using a digital fruit hardness tester (Model: FT-327, STEP Systems GmbH, Nuremberg, Germany) with a 6 mm cylindrical flat probe, following the puncture testing principle [23]. Prior to measurement, the instrument was calibrated according to the manufacturer’s protocol using standard weights to ensure measurement accuracy.

For the testing procedure, fruits of uniform size and maturity without visible surface defects were selected. Each fruit was positioned on the instrument’s stabilized platform with the blossom end facing downward. The probe was aligned perpendicularly to the fruit surface at the equatorial region. The piston was then driven into the fruit flesh at a constant speed of 2 mm/s until reaching a penetration depth of 10 mm, which was automatically controlled by the instrument. At the completion of penetration, the maximum force required, expressed in Newtons (N), was recorded as the FI value.

Two spatially separated measurement points, approximately 90–120 degrees apart along the equatorial plane, were tested for each fruit. The prism surface was meticulously cleaned with distilled water and softly wiped with lint-free tissue paper between measurements to prevent cross-contamination. The final FI value for each fruit was calculated as the arithmetic mean of the two measurements. A minimum of ten fruits per treatment group were analyzed to ensure statistical reliability. All measurements were conducted under controlled ambient conditions (20 ± 2 °C) to minimize temperature-induced variations.

#### 2.5.3. Soluble Solids Content (SSC)

The SSC of tomato fruits was determined using a digital handheld refractometer (PAL-1, Atago, Tokyo, Japan) following the manufacturer’s instructions and standard methodology [24]. The detailed procedure was as follows: Representative tomato fruits were selected and thoroughly washed with distilled water. After removing the surface moisture, the fruits were cut into quarters and homogenized using a commercial blender. The resulting pulp was immediately filtered through four layers of cheesecloth to obtain clear juice for analysis.

Prior to measurement, the refractometer was calibrated with distilled water to ensure zero-point accuracy. A sufficient volume of the freshly prepared tomato juice was applied to cover the entire surface of the measuring prism using a clean pipette. The instrument was then pointed toward a natural light source, and the measurement was initiated by pressing the start button. The soluble solids content, expressed as °Brix, was automatically displayed on the digital screen after temperature compensation.

Each tomato sample was analyzed in triplicate using independent juice preparations. Between measurements, the prism surface was meticulously cleaned with distilled water and softly wiped with lint-free tissue paper to prevent cross-contamination. The final SSC value for each sample was calculated as the mean of three replicate measurements. All determinations were conducted at ambient temperature (25 ± 2 °C) as specified in the instrument operating manual.

#### 2.5.4. Titratable Acid Content (TA)

The TA of tomato fruit was determined using alkaline titration, adapted from AOAC standard methods [25]. The following procedure was employed: Approximately 10.0 g of homogenized tomato flesh were accurately weighed into a mortar and thoroughly ground to a homogeneous pulp. The entire pulp was quantitatively transferred into a 100 mL volumetric flask using approximately 50 mL of freshly boiled and cooled deionized water for rinsing. The flask was then allowed to stand at room temperature for 30 min with intermittent shaking to facilitate extraction. After this period, the volume was made up to the mark with deionized water and mixed thoroughly. The mixture was subsequently filtered through a medium-flow qualitative filter paper, discarding the first 10 mL of the filtrate.

A 20.0 mL aliquot of the clear filtrate was pipetted into a 250 mL conical flask. To this, two drops of a 1% (*w*/*v*) phenolphthalein indicator solution in ethanol were added. The solution was then titrated with a pre-standardized 0.1 mol/L sodium hydroxide (NaOH) solution. Titration was performed preferably with magnetic stirring until a faint pink endpoint, persisting for not less than 30 s, was reached. The volume of the NaOH solution consumed was recorded. The entire process, from sample preparation to titration, was carried out in triplicate to ensure statistical reliability. The TA was calculated and expressed as a percentage of the predominant organic acid based on the volume and exact concentration of the NaOH used, the dilution factor, and the initial mass of the sample.

#### 2.5.5. Soluble Sugars (SS)

The SS in the samples was determined using the anthrone-sulfuric acid colorimetric method [26]. The detailed procedure was as follows: Exactly 0.2000 g of freshly prepared sample homogenate was weighed into a 15 mL stoppered centrifuge tube, followed by the addition of 8.0 mL of distilled water. The mixture was heated in a boiling water bath for exactly 30 min. After cooling to room temperature, the solution was filtered through medium-speed quantitative filter paper into a 50 mL volumetric flask. The residue was washed three times with 5 mL of distilled water, and the filtrates were combined and diluted to the mark. A 2.0 mL aliquot of the diluted solution was transferred to a stoppered colorimetric tube, mixed immediately with 4.0 mL of freshly prepared 0.2% anthrone-sulfuric acid solution (prepared by dissolving 0.20 g of anthrone in 100 mL of concentrated sulfuric acid), and heated in a boiling water bath for 10 min. After cooling in an ice-water bath, the absorbance of the reaction solution was measured at 630 nm using a UV-visible spectrophotometer (UV-2600, Shimadzu, Kyoto, Japan), with a distilled water blank subjected to the same treatment. All samples were analyzed in triplicate.

#### 2.5.6. Vitamin C Content (VC)

VC was determined using a commercial assay kit (Suzhou Keming Biotechnology Co., Ltd., Suzhou, China) based on the 2,6-dichlorophenolindophenol (DCIP) titration method, consistent with AOAC Official Method 967.21 [27].

Prior to sample analysis, the exact concentration of the DCIP titrant was determined through standardization. A 1.0 mL aliquot of a freshly prepared ascorbic acid standard solution (1.000 mg/mL, prepared by accurately dissolving 100.0 mg of L-ascorbic acid reference standard in 100 mL of 2% (*w*/*v*) oxalic acid solution) was pipetted into a 50 mL conical flask. Then, 10.0 mL of 2% (*w*/*v*) oxalic acid solution was added. This mixture was titrated immediately with the DCIP solution (nominal concentration 0.2 g/L) from a 50 mL burette. The titration was performed rapidly while gently swirling the flask, until the appearance of a faint pink color that persisted for at least 15 s. The volume of DCIP solution consumed was recorded. This standardization procedure was repeated in triplicate. The exact concentration of the DCIP solution (C_DCIP, in mg/mL) was calculated based on the equivalence that 1 mL of DCIP solution is reduced by 0.088 mg of ascorbic acid.

#### 2.5.7. Lycopene Content (LYC)

LYC was extracted and determined from tomato samples following the method of Nagata and Yamashita (1992) with minor modifications [28]. The specific procedure was as follows: Approximately 2.0 g (accurate to 0.1 mg) of homogenized tomato puree was precisely weighed into a 50 mL amber stoppered centrifuge tube. Then, 10 mL of pre-cooled anhydrous ethanol and 10 mL of anhydrous methanol were added sequentially. After each solvent addition, the mixture was vortexed for 2 min to remove water and polar interferents. Subsequently, 15 mL of petroleum ether (boiling range 30–60 °C) and 10 mL of 2% (*v*/*v*) dichloromethane solution were added. The mixture was shaken vigorously for 10 min for extraction, followed by centrifugation at 5000 rpm and 4 °C for 10 min. The upper red petroleum ether layer was carefully transferred to a 25 mL amber volumetric flask. The residue was then re-extracted with 10 mL of a mixed extraction solution (petroleum ether: anhydrous ethanol: anhydrous methanol = 1: 1: 2, *v*/*v*/*v*). The combined extracts were diluted to the mark with petroleum ether. The absorbance was immediately measured at 503 nm using a UV-visible spectrophotometer (UV-2600, Shimadzu, Japan) with a 1 cm pathlength quartz cuvette, using a mixture of petroleum ether and 2% dichloromethane as the reference blank. All samples were analyzed in triplicate.

### 2.6. Tomato Ripeness Grading Standards

According to the People’s Republic of China’s Supply and Marketing Cooperative Industry Standard “Tomato” (GH/T 1193-2021) [29], tomato maturity is classified into six stages: immature, green mature, color-changing, early red ripe, medium red ripe, and late red ripe. This study aims to non-destructively detect and predict tomato maturity and internal/external quality characteristics while the fruit is still on the plant. This information will be used to formulate precise harvesting strategies. The first grade of the six-grade maturity classification represents the immature stage. During this stage, the fruits have not fully developed and are difficult to ripen after harvesting. This makes them unsuitable for picking. Therefore, the first grade was excluded. Grades 2–6 of the six-grade system correspond exactly to grades 1–5 of the five-grade system, which are defined by the percentage of red surface area on the fruit’s skin. Therefore, the six-grade maturity classification was adjusted to a five-grade system (Table 3).

### 2.7. Parameter Configuration

Three models were developed using Python 3.7, PyCharm 2024.1.2, and PyTorch 1.8.1: an environmental prediction model, a tomato color prediction model, and a quality prediction model. The experimental hardware platform consisted of an Intel Core i5-9300H processor and an NVIDIA GeForce GTX 1650 graphics card. Systematic parameter optimization experiments determined the optimal training configurations for each model: The environmental prediction model used the Adam optimizer with a learning rate of 0.001, a batch size of 32, and 150 training epochs. The tomato color prediction model used the Adam optimizer with a learning rate of 0.001, an increased batch size of 64, and 150 training epochs. The quality prediction model used the Adam optimizer with a learning rate of 0.001, a batch size of 16, and 100 training epochs. After rigorous cross-validation and performance evaluation, these parameter configurations demonstrated optimal model prediction performance.

## 3. Environment Prediction Model Based on LSTM

### 3.1. LSTM (Long Short-Term Memory) Model

**Figure 5:**
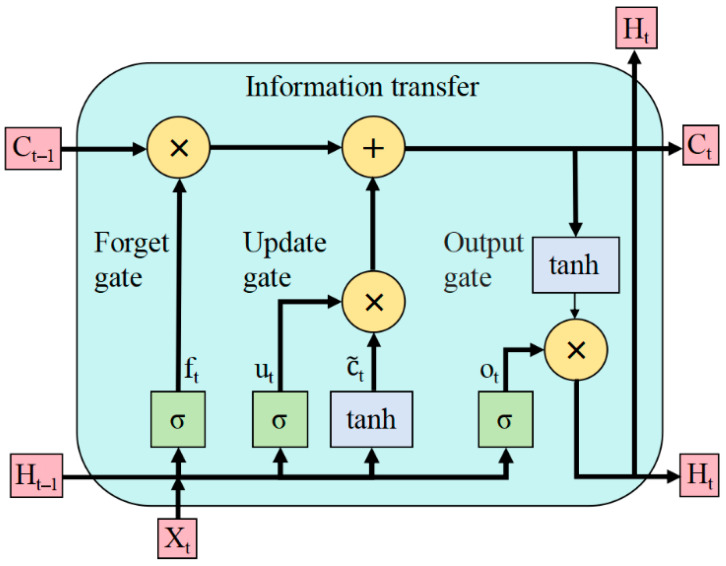
LSTM structure diagram.

Long Short-Term Memory (LSTM) networks are a specialized type of Recurrent Neural Network (RNN). They are designed to address the limitations of traditional RNNs, such as long-term dependency issues and gradient vanishing/exploding problems. This is achieved by introducing gating mechanisms [30]. Compared to standard RNNs, LSTMs demonstrate superior modeling capabilities for non-stationary time series with significant temporal dependencies and high noise levels [31,32]. The LSTM unit structure consists of the following key components (see Figure 5): forget gate (f_t_), update gate (u_t_), candidate cell state ( $\tilde{c}$_t_), and output gate (o_t_). These components work together through gating mechanisms to enable the dynamic selection, storage, and output of temporal information.

The working principle of the LSTM model is illustrated in Equations (8)–(13):

(8) $$f_{t}=\sigma \left(W_{f}\cdot \left[H_{t-1},X_{t}\right]+b_{f}\right)$$

(9) $$u_{t}=\sigma \left(W_{u}\cdot \left[H_{t-1},X_{t}\right]+b_{u}\right)$$

(10) $${\tilde{c}}_{t}=tanh\left(W_{c}\cdot \left[H_{t-1},X_{t}\right]+b_{c}\right)$$

(11) $$C_{t}=f_{t}\cdot C_{t-1}+u_{t}\cdot {\tilde{c}}_{t}$$

(12) $$o_{t}=\sigma \left(W_{o}\cdot \left[H_{t-1},X_{t}\right]+b_{o}\right)$$

(13) $$H_{t}=o_{t}\cdot \tanh\left(C_{t}\right)$$

In the above equations, *f_t_*, *u_t_*, ${\tilde{C}}_{t}$, *C_t_*, *O_t_*, and *H_t_* represent the forget gate, update gate, candidate cell state, current cell state, output gate, and hidden state, respectively. *W_f_*, *W_u_*, *Wc*, and *W_o_* denote the weight matrices of the forget gate, update gate, cell state, and output gate, respectively. *b_f_*, *b_u_*, *b_c_*, and *b_o_* represent the bias matrices of the forget gate, update gate, cell state, and output gate, respectively. tanh is the activation function and *σ* is the sigmoid activation function. *C_t−_*_1_ is the cell state at time t − 1. *C_t_* is the cell state at time t. *H_t_*_−1_ is the output at time t − 1. *H_t_* is the output at time t.

### 3.2. LSTM-Based Environmental Prediction Model

**Figure 6:**
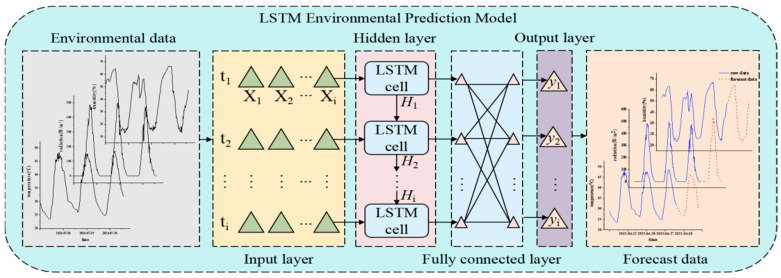
Structure diagram of the LSTM environmental prediction model.

This study proposes an environmental prediction model based on LSTM networks. The model uses raw time-series data directly to capture the temporal features and long-term dependencies of environmental parameters in greenhouses, such as temperature, humidity, and radiation. This enables the model to efficiently predict environmental parameters. Compared to traditional models, this method eliminates the need for complex preprocessing procedures, simplifies the model structure, and maintains strong prediction performance. Figure 6 shows the model’s overall architecture.

The LSTM-based environmental prediction model proposed in this study consists of an input layer, a hidden layer, a fully connected layer, and an output layer. The specific structure is as follows:

(1) Input Layer: Receives multidimensional environmental time series data, including air temperature, air humidity, and solar radiation. Converts the data into a three-dimensional tensor (*S*, *T*, *X*), which is suitable for long short-term memory (LSTM) processing. In this tensor, *S* represents the number of samples (96), *T* denotes the time step length (24), and *X* indicates the feature dimension (3).
(2) Hidden Layer: A multi-layer LSTM structure is used for temporal feature extraction and dependency modeling. Sequential information is transmitted through hidden states (*h*_1_~*h_i_*). The network’s depth and width are optimized for different environmental parameters.
  Air temperature prediction: Three LSTM layers, each with 100 hidden units;
  Air humidity prediction: One LSTM layer with 160 hidden units;
  Solar radiation prediction uses two LSTM layers, each with 64 hidden units.
(3) Fully Connected Layer: Integrates features extracted from the hidden layers and adjusts data dimensions to enhance the model’s expressive capability.
(4) Output Layer: Outputs the final predicted values of environmental parameters, accomplishing the time-series prediction task.

## 4. Tomato Ripeness Prediction Model Based on GRU-AT

### 4.1. GRU (Gated Recurrent Unit) Model

**Figure 7:**
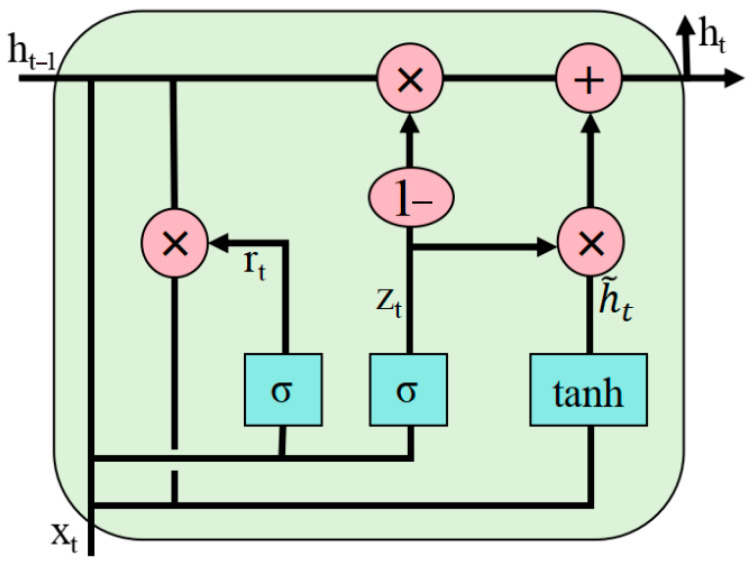
GRU structure diagram.

The Gated Recurrent Unit (GRU) is an efficient type of recurrent neural network [33] that has demonstrated significant advantages in time series modeling. Unlike the traditional LSTM network, the GRU uses a simplified dual-gate structure. The update gate regulates the integration of historical information and current inputs to maintain long-term dependencies in sequences. The reset gate controls the strength of the relationship between historical states and current observations [34]. This design reduces the number of model parameters and improves computational efficiency and convergence speed. Through its selective memory and forgetting mechanisms, the GRU effectively mitigates the vanishing and exploding gradient problems in recurrent neural networks [35]. Studies have shown that the GRU exhibits superior generalization performance on small-scale datasets [36], making it particularly suitable for time series analysis. By preserving the advantages of the LSTM network while reducing computational complexity, the GRU provides a more efficient solution for predicting time series data. Its structure is illustrated in Figure 7.

The formulas are shown in Equations (14)–(17):

(14) $$r_{t}=\sigma \left(w_{xr}x_{t}+w_{hr}h_{t-1}+b_{r}\right)$$

(15) $$z_{t}=\sigma \left(w_{xz}x_{t}+w_{hz}h_{t-1}+b_{z}\right)$$

(16) $${\tilde{h}}_{t}=\tanh\left(w_{xh}x_{t}+w_{hh}\left(r_{t}\cdot h_{t-1}\right)+b_{h}\right)$$

(17) $$h_{t}=z_{t}\cdot h_{t-1}+\left(1-z_{t}\right)\cdot {\tilde{h}}_{t}$$

*X_t_* represents the input at time step t, *h_t_* denotes the output at time step t, *h_t−_*_1_ refers to the output at time step t − 1, ${\tilde{h}}_{t}$ stands for the candidate hidden state, *w* and b are parameters to be trained by the model, *z* represents the update gate, *r* indicates the reset gate, *σ* denotes the sigmoid function, and tanh refers to the hyperbolic tangent function.

### 4.2. Attention Mechanisms

**Figure 8:**
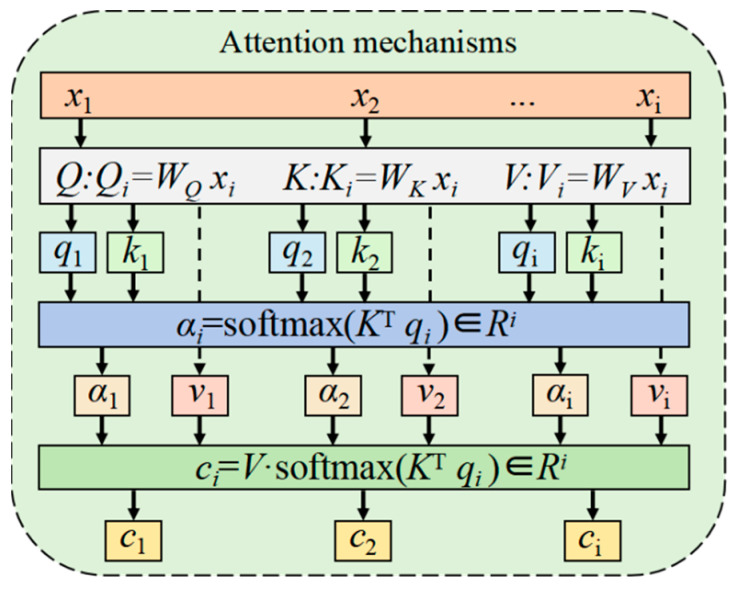
Attention mechanism.

The attention module (AT) [37] is a bio-inspired information processing mechanism whose core function is adaptive feature weighting via query-key-value (*Q*-*K*-*V*) computation. as depicted in Figure 8, the AT first applies linear transformations to the input features *x_i_* to generate a query vector *Q_i_* = *W_Q_x_i_*, a key vector *K_i_* = *W_K_x_i_*, and a value vector *V_i_* = *W_V_x_i_*. Next, attention weights *α_i_* are obtained by calculating the similarity between the query and key (*K^T^q_i_*), followed by softmax normalization. Finally, a context representation that emphasizes critical features is generated via weighted summation (*c_i_* = *V⋅α_i_*).

Its computational process is primarily described by the following Equations (18)–(22):

(18) $$Q:Q_{i}=W_{Q}x_{i}$$

(19) $$K:K_{i}=W_{K}x_{i}$$

(20) $$V:V_{i}=W_{V}x_{i}$$

(21) $$\alpha_{i}=softmax\left(K^{T}q_{i}\right)$$

(22) $$c_{i}=V\cdot softmax\left(K^{T}q_{i}\right)$$

Among these, *Q* represents the query vector, *K* denotes the key vector, and *V* signifies the value vector. *W_Q_*, *W_K_*, and *W_V_* are parameter matrices. *Q_i_* refers to an element of vector *Q*, *K_i_* to an element of vector *K*, and *V_i_* to an element of vector *V*. $\alpha_{i}$ represents the attention distribution, *c_i_* denotes the final output, and *i* indicates the feature index ranging from 1 to *n*.

### 4.3. GRU-AT Tomato Ripeness Prediction Model

**Figure 9:**
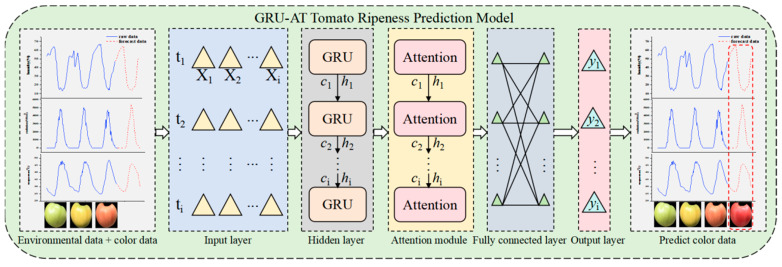
Structure diagram of the GRU-AT model.

This study addresses issues such as insufficient feature extraction and weak dynamic correlations in long-sequence prediction with traditional models by constructing a GRU-AT tomato ripeness prediction model consisting of GRU and AT modules. Figure 9 shows the model’s overall architecture.

The GRU-AT environmental prediction model consists of an input layer, a hidden layer, an AT, a fully connected layer, and an output layer. Its detailed structure is as follows:

(1) Input Layer: The model input consists of two types of time-series features collected simultaneously. Environmental parameters: Air temperature (°C), Relative humidity (%), Solar radiation (W/m^2^). Phenotypic features: Tomato fruit surface color percentages (proportions of red, yellow, and green channels). The input data is converted into a three-dimensional tensor (*S*, *T*, *X*) suitable for GRU processing.
  S denotes the number of samples (96);
  T represents the number of time steps (24);
  X indicates the number of features (6).
(2) Hidden Layer: The hidden layer is constructed using a GRU, which adaptively captures long-term dependencies in time-series data through update and reset gate mechanisms. The GRU’s computational process is defined by Equations (14) through (17), where zt and rt represent the update and reset gates, respectively, and ht denotes the hidden state at the current time step. The GRU layer outputs the hidden states of all time steps, {*h*_1_, *h*_2_, …, *h_t_*}, and transmits them to the attention module.
(3) Attention Module: This module is based on the attention mechanism and dynamically calculates the importance weights of hidden states at each time step. This enhances the feature contribution of key time steps. For the hidden state *h_t_* output by the GRU, the query (*Q*), key (*K*), and value (*V*) are computed as described in Equation (18) to (20). The similarity score between the query (*Q*) and the key (*K*) is calculated using the dot product in Equation (21). The attention weight (*α_t_*) is obtained using softmax normalization. The context feature representation, *c_t_*, is generated by summing and weighting the value vectors, *V*, with the attention weights, *α_t_*, as specified in Equation (22). This module significantly improves the model’s sensitivity to critical environmental changes and color variations by adaptively allocating weights.
(4) Fully Connected Layer: In this layer, the context features *c_t_* output by the attention module undergo nonlinear transformation and dimensionality mapping. This layer further integrates local features and enhances the model’s representational capacity, as described in Equation (23):
  (23) $$y_{FC}=ReLU\left(W_{FC}c_{t}+b_{FC}\right)$$
  where *y_FC_* denotes the output of the fully connected layer, *W_FC_* is a trainable parameter matrix used for feature space transformation, *c_t_* represents the context feature vector output by the attention module, and *b_FC_* is the bias vector of the fully connected layer.
(5) Output Layer: The final prediction result of the GRU-AT model is generated through a linear layer, as described in Equation (24):
  (24) $$\hat{y}=W_{o}\cdot y_{FC}+b_{o}$$
  where *W_o_* is the weight matrix of the output layer, *b_o_* denotes the bias term of the output layer, and $\hat{y}$ represents the output of the output layer.

## 5. Tomato Quality Prediction Model Based on Color Characteristics

### 5.1. Deep Neural Network (DNN) Model

**Figure 10:**
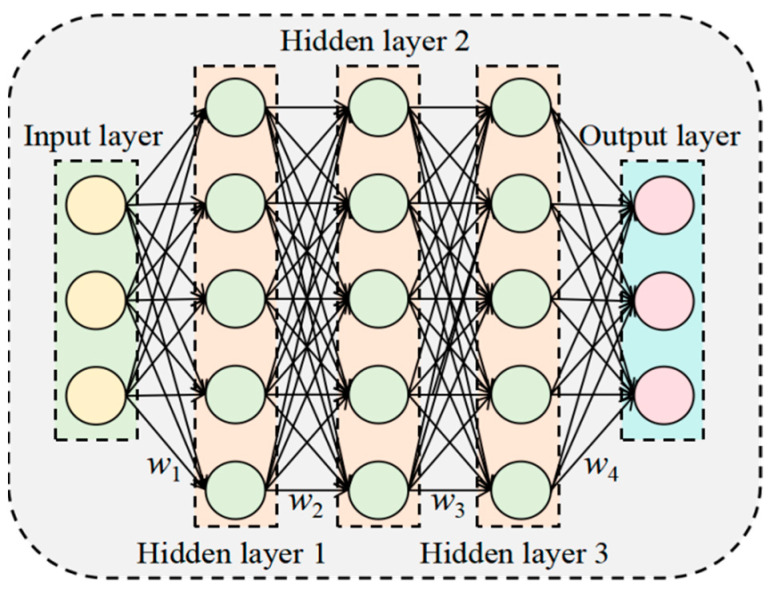
DNN Structure Diagram.

Deep neural network (DNN) are a type of feedforward network architecture that builds on multilayer perceptrons (MLPs). Their core structure consists of three fundamental components: an input layer, which receives raw data; an output layer, which outputs prediction results; and hidden layers, which learn and represent complex feature information in the data. The number of hidden layers is usually determined by the complexity of the problem. In this network, each neuron is connected to all the neurons in the previous layer. This section designs a five-layer, fully connected neural network comprising one input layer, three hidden layers, and one output layer. The input layer has the same number of neurons as the number of input feature parameters. The three hidden layers have 256, 128, and 64 neurons, respectively. The number of neurons in the output layer corresponds to the number of features to be predicted. Figure 10 illustrates the specific structure.

The formulas are shown in Equations (25)–(29):

(25) $$R\left(x\right)=\max\left(0,X\right)$$

(26) $$H_{1}=R\left(w_{1}\times X+b_{1}\right)$$

(27) $$H_{2}=R\left(w_{2}\times H_{1}+b_{2}\right)$$

(28) $$H_{3}=R\left(w_{3}\times H_{2}+b_{3}\right)$$

(29) $$Y=w_{4}\times H_{3}+b_{4}$$

Here, *R*(*x*) denotes the ReLU activation function. *X* represents the input features. *H*_1_, *H*_2_, and *H*_3_ correspond to the first, second, and third hidden layers, respectively. *w*_1_, *w*_2_, *w*_3_, and *w*_4_ are the weight matrices. *b*_1_, *b*_2_, *b*_3_, and *b*_4_ are the bias terms. *Y* denotes the output features.

### 5.2. A DNN Model for Tomato Quality Estimation

**Figure 11:**
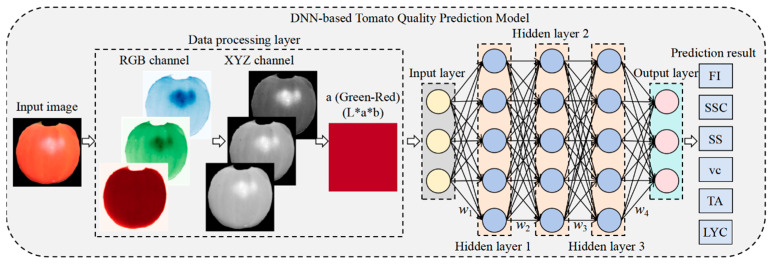
Structure Diagram of the DNN-Based Tomato Quality Prediction Model.

This study developed a tomato quality prediction model based on a DNN. The model establishes a nonlinear mapping relationship between the surface color features of tomatoes (the a value in the L*a*b color space) and internal and external quality parameters FI, SSC, SS, AT, VC, and LYC. This enables the non-destructive prediction of internal and external quality indicators, which were previously measurable only through destructive sampling using traditional methods, using only images. This effectively resolves issues associated with conventional detection methods, such as sample destruction and reliance on costly spectral equipment. Figure 11 shows the model’s structure.

The DNN-based tomato quality prediction model proposed in this study consists of a data processing layer, an input layer, hidden layers, and an output layer. The specific structure is as follows:

(1) Data Processing Layer: This layer is specifically responsible for converting images to the L*a*b* color space. It converts input RGB tomato images into the L*a*b* color space and extracts feature values from the *a* channel (green-red) as the primary input. It also normalizes the data to provide standardized feature inputs for subsequent networks. The specific formulas are presented in Equations (30)–(34):
  (30) $$C_{linear}=\left\{\begin{array}{cc} \frac{C}{12.92} & C\leq 0.04045\\ {\left(\frac{C+0.055}{1.055}\right)}^{2.4} & C>0.04045 \end{array}\right.$$
  (31) $$\left[\begin{array}{c} X\\ Y\\ Z \end{array}\right]=\left[\begin{array}{ccc} 0.412453 & 0.357580 & 0.180423\\ 0.212671 & 0.715160 & 0.072169\\ 0.019334 & 0.119193 & 0.950227 \end{array}\right]\left[\begin{array}{c} R_{linear}\\ G_{linear}\\ B_{linear} \end{array}\right]$$
  (32) $$a=500\left[f\left(\frac{X}{X_{n}}\right)-f\left(\frac{Y}{Y_{n}}\right)\right]$$
  (33) $$f\left(t\right)=\left\{\begin{array}{cc} t^{\frac{1}{3}} & t>\delta^{3}\\ \frac{t}{{3\delta }^{2}}+\frac{4}{29} & otherwise \end{array}\right.$$
  (34) $$a_{n}=\frac{a+128}{255}$$
  Here, *C* represents the input RGB channel value and *C_linear_* denotes the linearized RGB value. The weights for calculating the *X* component are [0.412453, 0.357580, 0.180423], and the weights for calculating the *Y* component are [0.212671, 0.715160, 0.072169]. represent the weights for calculating the *Z* component. *X*, *Y*, and *Z* represent the coordinates of the color in the CIE XYZ space. *R_linear_* represents the linear red channel value after inverse gamma correction transformation. *G_linear_* represents the linear green channel value after inverse gamma correction transformation. *B_linear_* represents the linear blue channel value after inverse gamma correction transformation. *a* represents the distribution of the color between red and green. *f*(*t*) is the nonlinear function, and a_n_ is the normalized value of the a channel.
(2) Input Layer: It receives the preprocessed a value features and ensures consistent data distribution through batch normalization processing. The input dimension is designed with one sample, laying the foundation for subsequent deep feature extraction.
(3) Hidden Layers: The hidden layers use a fully connected neural network structure with three layers, configured with 256, 128, and 64 neurons, respectively. All layers use the ReLU activation function to introduce nonlinear modeling capabilities. A dropout rate of 0.4 is applied between layers as a regularization strategy to prevent overfitting. This design, which progressively decreases the width of the network, effectively achieves layer-wise feature abstraction and compression.
(4) Output Layer: A multi-task regression architecture is used to predict the following six key tomato quality indicators directly: FI, SSC, SS, AT, VC, and LYC. All outputs use linear activation functions to generate actual measured values. To address the varying dimensional characteristics of the indicators, the model employs independent feature standardization processing and is optimized using an adaptively weighted, multi-task loss function [38]. During training, the loss weights of each sub-task are dynamically adjusted based on the respective indicator’s prediction error, enabling the model to accurately predict multiple physicochemical properties of tomatoes simultaneously. This approach enables end-to-end modeling, from single color features to comprehensive quality assessment.

This study uses three types of metrics to evaluate the prediction model’s performance in a systematic way. The coefficient of determination (R^2^) measures the model’s explanatory power. R^2^ reflects the model’s goodness of fit by calculating the proportion of variance in the target variable that the model explains. Its value ranges from -∞ to 1. An ideal value of 1 indicates a perfect fit. Mean absolute error (MAE) serves as a robustness indicator and reflects the magnitude of prediction errors by calculating the average absolute difference between predicted and true values. MAE is insensitive to outliers and shares the same unit of measurement as the target variable. Root mean square error (RMSE) amplifies the penalty for large errors by taking the square root of the mean squared error. RMSE is more sensitive to extreme values and effectively reflects prediction accuracy. These three metrics assess the model’s predictive performance comprehensively from different dimensions. Their calculation formulas are shown in Equations (35)–(37):

(35) $$R^{2}=1-\frac{\sum_{i=1}^{N}\left(y^{\prime }-y\right)}{\sum_{i=1}^{N}\left(y-\bar{y}\right)}$$

(36) $$MAE=\frac{1}{N}\sum_{i=1}^{N}\left|y^{\prime }-y\right|$$

(37) $$RMSE=\sqrt{\frac{1}{N}\sum_{i=1}^{N}\left(y^{\prime }-y\right)}$$

Note that *y* denotes the true value, *ŷ* denotes the model-predicted value, and *ȳ* denotes the mean of the true values.

## 6. Results

### 6.1. Investigation Into the Performance Comparison of Environmental Prediction Models

**Figure 12:**
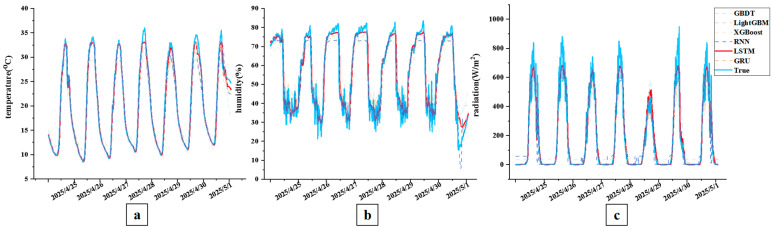
Comparison of environmental prediction model performance curves. Note: (**a**) shows the comparison of temperature predictions among different models; (**b**) shows the comparison of humidity predictions among different models; (**c**) shows the comparison of radiation predictions among different models.

**Figure 13:**
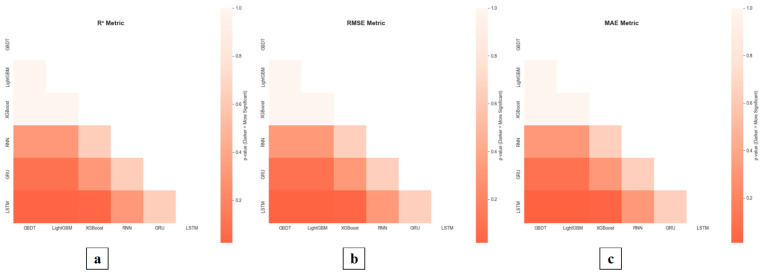
Nemenyi Post hoc Test—Statistical Significance (α = 0.05) (Environment). Note: (**a**) shows the comparison of the R^2^ metric among different models; (**b**) shows the comparison of the RMSE metric among different models; (**c**) shows the comparison of the MAE metric among different models.

This study conducted comparative experiments with classical models, including GBDT, LightGBM, XGBoost, RNN, and GRU, to validate the LSTM model’s effectiveness in environmental prediction tasks. The LSTM model demonstrated significant advantages across multiple evaluation metrics in the predictive performance analysis of three key environmental indicators: temperature, humidity, and radiation, the results are presented in Table 4 and Table 5 and Figure 12 and Figure 13.

#### 6.1.1. Validation of the Superiority of the LSTM Model in Temperature Prediction

In the temperature prediction task, the LSTM model demonstrated exceptional performance, achieving an R^2^ of 0.9931. Statistical analysis established its superiority over all tree-based models (GBDT, LightGBM, XGBoost) with significance (*p* < 0.05), while no significant difference was observed when compared to other deep learning models (GRU and RNN). Specifically, its RMSE and MAE were 0.7016 and 0.4115, respectively, representing reductions of 35.2% and 31.9% compared to the second-best performing GRU model. The advantage over traditional methods like XGBoost was even more pronounced in terms of RMSE. These results confirm that LSTM’s gating mechanisms and memory cell architecture enable it to effectively capture long-term dependencies in temperature sequences, achieving superior prediction accuracy.

#### 6.1.2. Validation of the Superiority of the LSTM Model in Humidity Prediction

In the humidity prediction task, despite the presence of noise interference and highly nonlinear characteristics, the LSTM model still demonstrated exceptional performance. The model achieved an R^2^ value of 0.9559, performing better than both GRU and RNN models. In terms of the MAE metric, the LSTM model reached 2.8500, which is lower than that of the GRU model and also shows a quantifiable advantage over tree-based models such as GBDT (3.3049). Meanwhile, the LSTM model’s RMSE was as low as 4.1167—the best result among all comparative models—fully validating its exceptional feature extraction capability and anti-interference performance in complex humidity sequences.

#### 6.1.3. Validation of the Superiority of the LSTM Model in Radiation Prediction

Even for the highly volatile and difficult-to-predict radiation sequences, the LSTM model maintained a competitive advantage, achieving the highest R^2^ value of 0.9609 among all compared models. Statistical results indicated that although this R^2^ value did not show a significant difference from the GRU model (*p* = 0.645), it demonstrated a significant advantage over the RNN model (*p* = 0.045). In terms of RMSE and MAE, the LSTM model achieved the best values of 50.4698 and 27.0234, respectively, significantly outperforming all tree-based models (GBDT: *p* = 0.011; LightGBM: *p* = 0.011; XGBoost: *p* = 0.023). These results confirm that the LSTM model maintains robust temporal modeling capabilities and stable predictive performance, even when processing highly fluctuating radiation sequences with substantial noise interference.

Based on the prediction results of three environmental indicators, the LSTM model demonstrated statistically significant advantages over traditional tree-based machine learning methods (GBDT, LightGBM, XGBoost) across all key performance metrics (R^2^, RMSE, MAE), while maintaining a leading position among deep learning models. The model’s unique gating mechanism and powerful sequence modeling capabilities enabled it to effectively handle long-term dependencies and mitigate noise interference across different environmental variables, thereby validating its robust performance and practical value in environmental prediction tasks.

### 6.2. A Study on the Performance Comparison of Tomato Maturity Prediction Models

**Figure 14:**
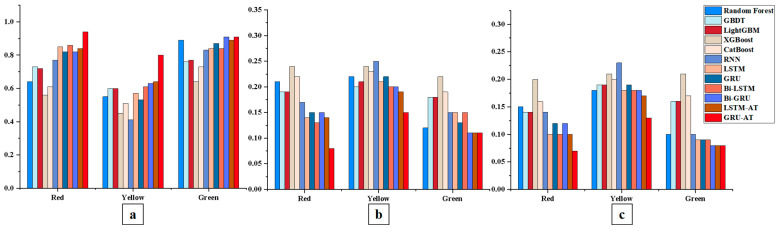
Comparison of tomato ripeness prediction model performance. Note: (**a**–**c**) present the comparison of R^2^, RMSE, and MAE performance metrics, respectively, across the different models.

**Figure 15:**
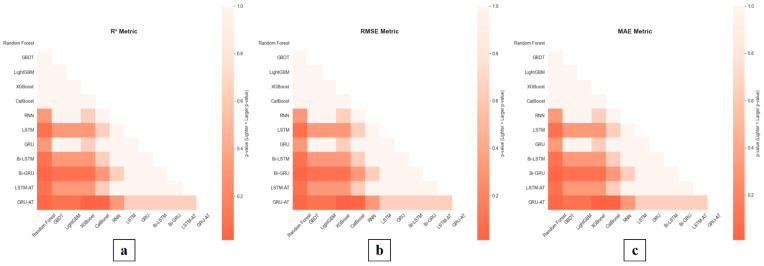
Nemenyi Post hoc Test—Statistical Significance (α = 0.05) (Maturity). Note: (**a**) shows the comparison of the R^2^ metric among different models; (**b**) shows the comparison of the RMSE metric among different models; (**c**) shows the comparison of the MAE metric among different models.

Systematic comparisons were made with traditional models, including Random Forest, GBDT, LightGBM, XGBoost, CatBoost, RNN, LSTM, GRU, Bi-LSTM, and Bi-GRU, to validate the GRU-AT model’s effectiveness in tomato maturity prediction. During the experiments, environmental data predicted by the environmental model was used as input for the maturity prediction model to forecast tomato maturity. The results demonstrate that the GRU-AT model has significant advantages in prediction accuracy and stability. See Table 6 and Table 7 and Figure 14 and Figure 15 for details.

#### 6.2.1. A Comparative Evaluation of the Red Proportion Prediction Performance of the GRU-AT Model and Classical Models

In the red proportion prediction task, the GRU-AT model demonstrated superior performance, achieving a coefficient of determination (R^2^) of 0.94. This represents a 14.6% improvement over the standard GRU model (R^2^ = 0.82) and a significant 28.8% improvement over the top-performing classical machine learning model, GBDT (R^2^ = 0.73). Regarding prediction errors, the GRU-AT model achieved an MAE of 0.07 and an RMSE of 0.08. Compared to the best-performing baseline sequential model, LSTM (MAE = 0.10, RMSE = 0.14), this corresponds to a 30% reduction in MAE and a 42.9% reduction in RMSE.

These results indicate that the integrated attention mechanism effectively enhances the model’s sensitivity to temporal dynamics in red maturity by dynamically focusing on features from critical time steps.

#### 6.2.2. A Comparative Evaluation of the Yellow Proportion Prediction Performance of the GRU-AT Model and Classical Models

In the yellow proportion prediction task, the GRU-AT model achieved an R^2^ of 0.80 and an MAE of 0.13. This represents a 27.0% improvement in R^2^ and a 35.0% reduction in MAE compared to the CatBoost model (R^2^ = 0.63, MAE = 0.20). Notably, the GRU-AT model’s MAE decreased by 27.8% compared to the Bi-GRU model (MAE = 0.18), validating the attention mechanism’s superior ability to capture critical temporal features in the color gradient process.

#### 6.2.3. A Comparative Evaluation of the Green Proportion Prediction Performance of the GRU-AT Model and Classical Models

In the green proportion prediction task, the GRU-AT model demonstrated stable performance, achieving a coefficient of determination (R^2^) of 0.91, which is comparable to the best-performing baseline model Bi-GRU (R^2^ = 0.91). The model attained a mean absolute error (MAE) of 0.08, representing a 50.0% reduction compared to the LightGBM model (MAE = 0.16). These results indicate that the attention mechanism effectively optimizes feature weight allocation during color transition processes. Furthermore, the GRU-AT model shows improved prediction accuracy for green proportion compared to traditional machine learning models, as evidenced by the performance advantage over XGBoost (R^2^ = 0.64, MAE = 0.21).

Experimental results demonstrate that the GRU-AT model exhibits statistically significant advantages in tomato ripeness prediction. By incorporating an attention mechanism, the model effectively captures critical temporal features during the ripening process, surpassing traditional models in both prediction accuracy and stability. Specifically, the GRU-AT model achieved an R^2^ of 0.94, showing significant improvements over the best-performing traditional machine learning model GBDT (R^2^ = 0.73, *p* = 0.015) and the standard GRU model (R^2^ = 0.82, *p* = 0.047). In terms of prediction errors, the GRU-AT model attained an MAE of 0.07 and RMSE of 0.08, representing statistically significant reductions compared to the LSTM model (MAE = 0.10, *p* = 0.094; RMSE = 0.14, *p* = 0.094). In the most challenging yellow proportion prediction task, the GRU-AT model achieved an MAE of 0.13, demonstrating significant improvement over the traditional RNN model (MAE = 0.23, *p* = 0.317) and notable advantage over the CatBoost model (MAE = 0.20, *p* = 0.094).

Statistical analysis further confirmed that the GRU-AT model’s performance advantages over tree-based models (Random Forest, GBDT, LightGBM, XGBoost, CatBoost) reached high significance levels across all three evaluation metrics (*p* < 0.05 in most comparisons). Particularly during color transition phases, the GRU-AT model demonstrates significantly reduced prediction error fluctuations (*p* < 0.05) and substantially improved alignment between prediction curves and actual observations.

These results confirm that the attention mechanism effectively enhances the model’s feature extraction capability for color gradient processes while significantly improving prediction reliability and stability, providing an effective technical solution for accurate tomato ripeness prediction.

### 6.3. Performance Analysis of Tomato Quality Prediction Models

This study developed a deep learning-based multi-task quality prediction model to investigate the intrinsic relationship between tomato surface color (i.e., ripeness) and internal quality. The model uses ripeness, which characterizes surface color, as a key input and leverages deep neural networks to uncover relationships with core quality parameters, such as FI, SSC, SS, TA, VC, and LYC. This allows for the quantitative, non-destructive prediction of tomato quality based on visual features.

**Figure 16:**
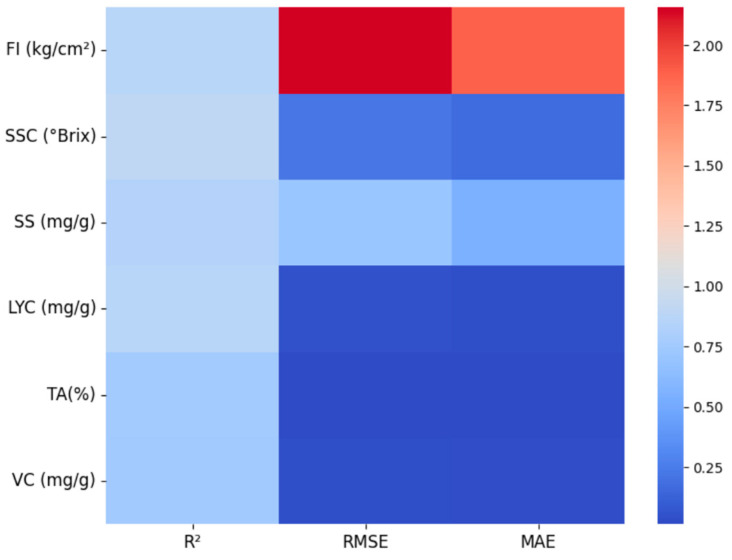
Heat map of performance indicators for tomato quality prediction.

As shown in Table 8 and Figure 16, the DNN model developed in this study demonstrated excellent and statistically significant performance in predicting multiple tomato quality indicators. For FI prediction, the model achieved an R^2^ of 0.8709 (*p* = 0.031) with an MAE of 1.8859 kg/cm^2^, indicating statistically significant and reliable predictive capability for mechanical properties. The model performed particularly well in predicting SSC (°Brix), attaining an R^2^ of 0.9061 (*p* = 0.019) and an RMSE of 0.2157 °Brix, reflecting highly significant and precise sugar content prediction ability.

Statistical test results revealed that the model demonstrated significant performance across four core quality indicators: SS prediction achieved an R^2^ of 0.8352 (*p* = 0.045), while LYC prediction reached an R^2^ of 0.8719 (*p* = 0.031). Although the R^2^ values for TA and VC predictions (0.7557 and 0.7485, respectively) did not reach statistical significance (*p* > 0.05), their MAE values remained at relatively low levels of 0.0140% and 0.0269 mg/g, respectively, demonstrating stable prediction trends.

These results indicate that the DNN model can accurately and comprehensively predict multiple key tomato quality indicators, exhibiting statistically superior performance particularly in core metrics such as SSC, FI, and LYC, thereby providing reliable technical support for intelligent quality assessment of tomatoes.

### 6.4. Performance Analysis of the Integrated Tomato Quality Prediction System

**Figure 17:**
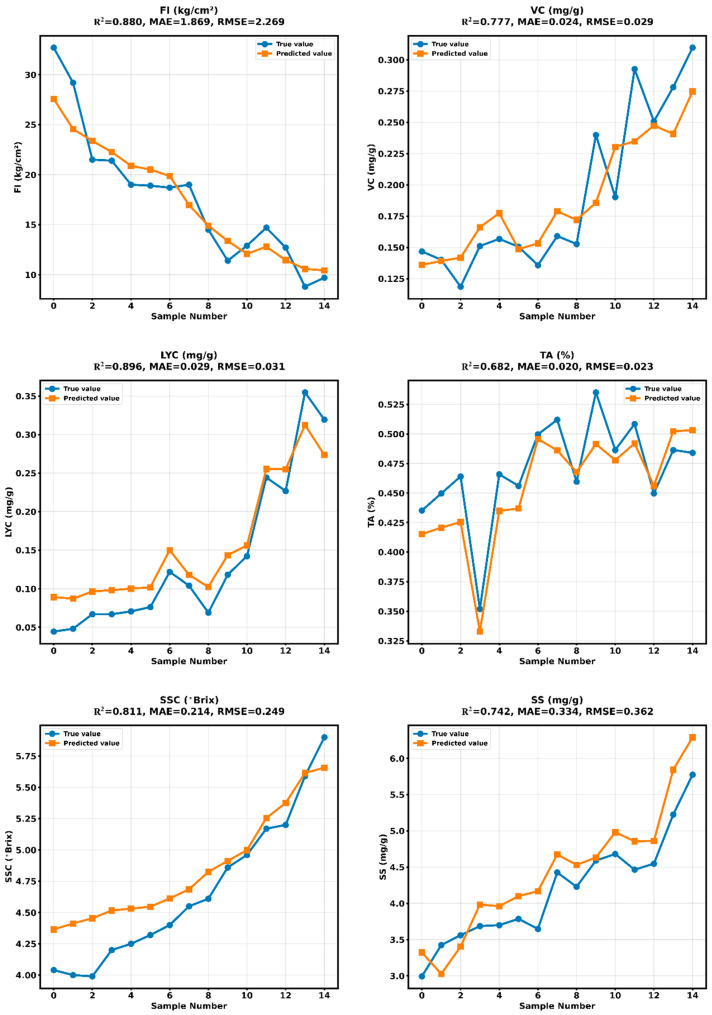
Comparison of the prediction performance curves of the tomato quality prediction model.

Based on the tomato quality prediction model system developed in this study, the experimental results shown in Figure 17 are analyzed as follows. This integrated system consists of an environmental prediction LSTM model, a maturity prediction GRU-AT model, and a quality prediction DNN model. By comprehensively evaluating the predictive performance of various quality indicators (FI, SSC, TA, VC, and LYC) derived from image data, we have thoroughly assessed the system’s overall performance, key technological innovations, and potential application value in facility-based tomato production.

The multi-model system exhibits excellent performance in predicting major tomato quality parameters, confirming its effectiveness. For FI prediction, the system attained an R^2^ of 0.880 with MAE of 1.869 kg/cm^2^ and RMSE of 2.269 kg/cm^2^, indicating reliable mechanical property assessment capability. The system performed particularly well in LYC content prediction, achieving an R^2^ of 0.896 with MAE of 0.029 mg/g and RMSE of 0.031 mg/g. The prediction of SSC was also accurate, with an R^2^ of 0.811, an MAE of 0.214 °Brix, and an RMSE of 0.249 °Brix.

Although VC prediction showed moderate explanatory power (R^2^ = 0.777), it maintained low error rates (MAE = 0.024 mg/g, RMSE = 0.029 mg/g). Similarly, despite relatively lower R^2^ values for TA and S5 predictions (0.682 and 0.742, respectively), they consistently maintained low error levels, demonstrating the system’s stable predictive capability across all quality indicators.

The integration of computer vision with deep learning architectures enables comprehensive quality assessment without destructive sampling. This technical approach represents a significant breakthrough in non-destructive quality monitoring for controlled environment agriculture, providing substantial support for precision management decisions throughout the entire growth cycle of tomatoes in facility-based production systems.

## 7. Discussion

This study developed an integrated end-to-end non-destructive tomato quality prediction model that combined three key modules: environmental forecasting, dynamic ripeness discrimination, and internal/external quality parameter estimation. Compared to traditional methods relying on expensive spectroscopic instruments or destructive sampling, our system requires only standard RGB images coupled with environmental sensor data to achieve accurate non-destructive prediction of key tomato quality indicators (FI, SSC, SS, TA, VC, and LYC).

The DNN model demonstrated excellent predictive performance for most quality indicators, particularly for LYC (R^2^ = 0.896), SSC (R^2^ = 0.811), and FI (R^2^ = 0.849). This indicates that reliable nonlinear mapping relationships have been established between the fruit surface color (a* value) and LYC, SSC, and FI. Although the predictive accuracy for TA(R^2^ = 0.682) and VC (R^2^ = 0.776) was relatively limited, the mean absolute error remained consistently low (TA: 0.0203%; VC: 0.0238 mg/g), confirming the model’s practical utility in controlling absolute error.

The innovation of this research is primarily reflected in three aspects: First, the proposed “environment–phenotype–quality” tripartite coupling model framework systematically analyzes the synergistic mechanisms between light-temperature accumulation effects and dynamic fruit color evolution as well as internal quality formation. Second, the introduced attention-enhanced GRU network significantly improved temporal sensitivity in color ratio prediction by focusing on key maturation stages. Third, the constructed multi-task DNN regression model based on a single color feature (a* value) enabled simultaneous prediction of multiple quality indicators while substantially reducing hardware dependency and computational complexity.

The established correlations between color features and quality parameters are grounded in solid biological foundations. The strong association between the a* value and LYC reflects carotenoid biosynthesis during fruit maturation, while its relationship with SSC involves the accumulation of photosynthetic products and sugar metabolism processes. The model’s capability to capture these relationships not only provides technical solutions but also offers biological insights into quality formation patterns. For agricultural practitioners, this technology serves as a practical tool to optimize tomato production. By enabling non-destructive monitoring of fruit quality development, farmers can accurately determine optimal harvest timing and implement targeted cultivation management, ultimately enhancing both yield quality and economic returns.

### Limitations and Comparison with State-of-the-Art

While the proposed model demonstrates practical utility, particularly for color-associated traits, a transparent comparison with advanced non-destructive techniques is crucial for an objective assessment of its performance. As indicated by the results, the model’s predictive accuracy for TA and VC is limited (with coefficients of determination R^2^ of 0.682 and 0.777, respectively). This performance gap becomes more evident when directly compared against state-of-the-art methods. For instance, Zhao et al. (2023) [39] employed hyperspectral imaging combined with a CNN model, achieving superior predictive accuracy for TA (R^2^ = 0.87, RMSE = 0.03) compared to this study, as detailed in Table 9.

The performance disparity can be primarily attributed to a fundamental methodological limitation in our approach: the reliance on a single color feature (a value). Since the accumulation and degradation of TA and VC are regulated by multi-pathway metabolic mechanisms [40,41], their direct correlations with color changes are significantly weaker than those of SSC and LYC. In contrast, hyperspectral imaging captures hundreds of spectral bands containing rich molecular vibration and absorption information that is directly related to these biochemical compounds. This enables models such as CNNs to learn features that are inherently more predictive of acidity and vitamin content than color features alone.

This comparison directly illustrates the inherent trade-off between cost and accuracy that is central to our study. The high-performance system reported by Zhao et al. (2023) [39] represents the pinnacle of prediction accuracy but relies on expensive hyperspectral equipment and computationally intensive models, making it suitable for laboratory or high-value precision agriculture. Our system, by strategically limiting the input to a single, easily extractable feature, explicitly sacrifices this peak accuracy to achieve drastically lower hardware costs and computational complexity. This design choice makes the technology accessible for the intended end-users: ordinary farmers.

Therefore, the key question is not whether our model outperforms the state-of-the-art in absolute accuracy, but whether its accuracy is sufficient for its intended practical application. For in-field maturity screening and basic quality grading—where the goal is to categorize fruits into broad quality tiers rather than provide laboratory-grade measurements—the achieved performance, coupled with relatively low prediction errors (RMSE of 0.02% for TA), can be considered fit-for-purpose. The model successfully identifies trends and significant differences, which is often all that is required for harvest decision support.

Other limitations persist. Models developed based on a single variety (‘Provence’) under specific greenhouse conditions may experience performance fluctuations when applied to different varieties, cultivation modes, or climatic regions due to the genotype-dependent and environment-specific nature of color-quality correlations. Furthermore, although color calibration measures were adopted, the model remains susceptible to variations in lighting conditions, camera parameters, and shooting angles, with this sensitivity being particularly evident for indicators like TA and VC that weakly correlate with color features.

While the proposed model demonstrates robust performance within the context of this study, it is crucial to delineate its scope. The model was developed and validated exclusively on the ‘Provence’ tomato variety cultivated under specific greenhouse conditions. Consequently, its generalizability to other cultivars, production systems (e.g., open-field), or divergent climatic regions cannot be directly assumed. The correlations between color features and internal quality are known to be influenced by genotype-specific traits and environmental interactions. To address this limitation and enhance the model’s broad applicability, future work will prioritize external validation. This includes plans for cross-variety trials encompassing distinct tomato types and multi-site evaluations across different geographical locations and cultivation practices.

In summary, we acknowledge that the model’s simplicity imposes a performance ceiling, especially for biochemically complex traits. However, this limitation is a deliberate consequence of our design philosophy to prioritize affordability and deployability. Future work will focus on enhancing model generalization capability by exploring adaptability across multiple varieties and environments; improving data acquisition robustness through multi-light compensation or high dynamic range imaging techniques; deepening theoretical understanding of color-quality correlation mechanisms; and promoting system integration with edge computing devices to achieve real-time field diagnosis and decision-making applications.

## 8. Conclusions

This study addresses the core challenges of facility-based tomato production. Traditional quality detection methods are destructive and time-consuming, and existing non-destructive techniques, such as near-infrared spectroscopy, are costly and difficult to use in capturing the dynamic formation process of quality traits. To overcome these limitations, we developed an integrated, computer vision-driven framework combining environmental prediction, ripeness discrimination, and quality assessment. This framework can accurately predict key internal and external quality parameters of tomatoes non-destructively using only time-series RGB images. The main conclusions of this study are as follows:

A high-precision environmental prediction model was constructed to lay the data foundation for quality prediction. The developed LSTM model addressed the multi-factor coupling and nonlinear characteristics of greenhouse environments and demonstrated exceptional performance in predicting temperature (R^2^ = 0.9931), humidity (R^2^ = 0.9559), and radiation (R^2^ = 0.9609). It significantly outperformed comparative models, such as GBDT, XGBoost, RNN, and GRU. The model effectively captured the temporal evolution patterns of light and temperature conditions, providing reliable data for the subsequent analysis of the coupling relationships between environmental cumulative effects and fruit phenotype and quality.

We proposed a GRU-AT tomato ripeness dynamic prediction model that precisely models color evolution over time. Integrating predicted environmental data and real-time image color features allowed the model to effectively capture nonlinear and nonstationary changes during the maturation process. An attention mechanism was introduced to dynamically enhance feature extraction during critical color transition stages. This significantly improved the accuracy (R^2^ = 0.97) and stability of predicting the proportions of red, yellow, and green. Compared to 10 other models, including Random Forest, XGBoost, and LSTM, the model outperformed them all, providing an effective tool for elucidating the spatiotemporal coupling mechanism between dynamic color evolution and internal compositional changes.

We established a DNN-based quantitative prediction model for tomato quality, which validated the intrinsic “color-quality” relationship. The study confirmed a strong nonlinear mapping between tomato surface color (a-value in the Lab color space) and core internal quality indicators. The DNN model successfully predicted multiple quality parameters simultaneously using only color features from images, demonstrating particularly outstanding performance for LYC (R^2^ = 0.896), FI (R^2^ = 0.880), and SSC (R^2^ = 0.811, °Brix). While the prediction accuracy for titratable TA and VC was relatively lower, MAE remained low, indicating the model’s practical utility. These results demonstrate the technical feasibility of non-destructively assessing tomato internal quality using standard RGB images.

In summary, the methodological framework established in this study effectively integrates the complete pathway from environmental drivers to phenotypic responses and quality formation. This achieves substantial progress at three levels: First, by constructing an LSTM-based environmental prediction model, the cumulative effects of environmental factors, such as temperature, humidity, and radiation, on the fruit ripening process were quantitatively analyzed. This provided data to support an understanding of the relationship between external conditions and maturity. Second, using GRU-AT and DNN models, nonlinear mapping relationships were successfully established between multi-scale color features and internal/external quality traits, revealing the co-evolutionary pathway of appearance phenotypes and intrinsic quality. Third, the computer vision prediction system, which integrates multi-source data, accurately predicts tomato quality parameters based on time-series images. This offers a practical technical solution for non-destructive quality monitoring and harvest decision-making. Compared to traditional methods that rely on expensive spectroscopic equipment, the computer vision approach adopted in this study significantly reduces hardware costs and technical barriers, thus enhancing its applicability in real production environments. This provides a new technical option for smart tomato production in controlled environments. Future research could focus on integrating this technology with intelligent agricultural machinery to develop precision harvesting robotic systems with real-time perception, thereby advancing the transition of smart fruit and vegetable production from information sensing to intelligent execution.

## Figures and Tables

**Table 1:** Technical specifications of sensors.

Measurement Parameters	Model	Resolution	Accuracy	Measurement Range
Temperature sensor	SHT41	0.01 °C	±0.2 °C	−30 °C~70 °C
Humidity sensor	SHT41	0.01% RH	±2% RH	0~100% RH
radiation sensor	ISL89013	1 W/m^2^	±5%	0~1800 W/m^2^

**Table 2:** Image data distribution.

Maturity	Number of Images	Percentage
green ripening period	626	39.0%
transition period	266	16.6%
early ripening stage	166	10.3%
mid-ripening stage	146	9.1%
late ripening stage	402	25.0%
Total	1606	100%

**Table 3:** Tomato ripeness grading criteria.

Level	Maturity	RGB Image	Description
1	green ripening period	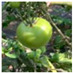	From green to white-green, can be artificially ripened, picked, and stored.
2	transition period	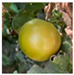	During the green-to-red transition, yellow or pale red spots emerge near the stem, with less than 10% red coverage.
3	early ripening stage	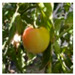	10% to 30% red ripeness, with 10% to 30% of the fruit surface turning red.
4	mid-ripening stage	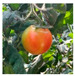	40% to 60% ripe, with 40% to 60% of the fruit surface red
5	late ripening stage	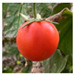	70% to 100% red ripeness, with 70% to 100% of the fruit surface turning red.

**Table 4:** Statistical comparison of environmental prediction model performance.

Model	Environmental Indicators	R^2^	RMSE	MAE
GBDT	temperature	0.9368	2.1234	1.3181
	humidity	0.9305	5.1698	3.3049
	radiation	0.9096	76.7382	39.7951
LightGBM	temperature	0.9356	2.1438	1.7724
	humidity	0.9086	5.9273	4.5531
	radiation	0.9360	64.5849	47.7284
XGBoost	temperature	0.9398	2.0720	1.7980
	humidity	0.9333	5.0633	4.6399
	radiation	0.8990	81.1269	44.3330
RNN	temperature	0.9738	1.3670	0.8302
	humidity	0.9462	4.5493	3.5779
	radiation	0.9267	69.0893	37.5481
GRU	temperature	0.9836	1.0825	0.6041
	humidity	0.9540	4.2072	2.9423
	radiation	0.9572	52.7824	21.5273
LSTM	temperature	0.9931	0.7016	0.4115
	humidity	0.9559	4.1167	2.8500
	radiation	0.9609	50.4698	27.0234

**Table 5:** Friedman Test Results Summary (Environment).

Performance Metric	Friedman χ^2^	Degrees of Freedom	*p*-Value	Significance (α = 0.05)
R^2^	13.000	5	0.023	Significant
RMSE	13.600	5	0.018	Significant
MAE	14.800	5	0.011	Significant

**Table 6:** Statistical comparison of tomato ripeness prediction model performance.

Model	Color	R^2^	RMSE	MAE
Random Forest	Red	0.64	0.21	0.15
	Yellow	0.55	0.22	0.18
	Green	0.89	0.12	0.10
GBDT	Red	0.73	0.19	0.14
	Yellow	0.60	0.20	0.19
	Green	0.76	0.18	0.16
LightGBM	Red	0.72	0.19	0.14
	Yellow	0.60	0.21	0.19
	Green	0.77	0.18	0.16
XGBoost	Red	0.56	0.24	0.20
	Yellow	0.45	0.24	0.21
	Green	0.64	0.22	0.21
CatBoost	Red	0.61	0.22	0.16
	Yellow	0.51	0.23	0.20
	Green	0.73	0.19	0.17
RNN	Red	0.77	0.17	0.14
	Yellow	0.41	0.25	0.23
	Green	0.83	0.15	0.10
LSTM	Red	0.85	0.14	0.10
	Yellow	0.57	0.21	0.18
	Green	0.84	0.15	0.09
GRU	Red	0.82	0.15	0.12
	Yellow	0.53	0.22	0.19
	Green	0.87	0.13	0.09
Bi-LSTM	Red	0.86	0.13	0.10
	Yellow	0.61	0.20	0.18
	Green	0.84	0.15	0.09
Bi-GRU	Red	0.82	0.15	0.12
	Yellow	0.63	0.20	0.18
	Green	0.91	0.11	0.08
LSTM-AT	Red	0.84	0.14	0.10
	Yellow	0.64	0.19	0.17
	Green	0.89	0.11	0.08
GRU-AT	Red	0.94	0.08	0.07
	Yellow	0.80	0.15	0.13
	Green	0.91	0.11	0.08

**Table 7:** Friedman Test Results Summary (Maturity).

Performance Metric	Friedman χ^2^	Degrees of Freedom	*p*-Value	Significance (α = 0.05)
R^2^	17.636	11	0.014	Significant
RMSE	18.182	11	0.011	Significant
MAE	18.727	11	0.009	Significant

**Table 8:** Statistical Significance Analysis of Prediction Performance Across Six Quality Indicators.

Quality Indicator	R^2^	F-Statistic	*p*-Value	Significance (α = 0.05)
FI (kg/cm^2^)	0.8709	13.50	0.031	Significant
SSC (°Brix)	0.9061	19.30	0.019	Significant
SS (mg/g)	0.8352	10.11	0.045	Significant
LYC (mg/g)	0.8719	13.57	0.031	Significant
TA (%)	0.7557	6.19	0.087	Not Significant
VC (mg/g)	0.7485	5.96	0.092	Not Significant

**Table 9:** Performance Comparison Between This Study and Zhao et al. (2023) [39].

Model	Quality Indicator	R^2^	RMSE
This study	FI (kg/cm^2^)	0.88	2.27
Zhao et al., 2023		0.92	0.94
This study	SSC (°Brix)	0.81	0.25
Zhao et al., 2023		0.88	0.19
This study	LYC (mg/g)	0.90	**0.03**
Zhao et al., 2023		0.94	0.73
This study	TA (%)	0.68	0.02
Zhao et al., 2023		**0.87**	0.03

## Data Availability

The data supporting the findings of this study are not publicly available due to their classification as confidential. The datasets were collected from border areas in Kashgar, Xinjiang, China, where environmental data are deemed sensitive and restricted by authorities.

## Abbreviations

The following abbreviations are used in this manuscript:

LSTM	Long Short-Term Memory
GRU	Gated Recurrent Unit
DNN	Deep Neural Network
AT	Attention Mechanism
FI	Firmness
SS	Soluble sugars
TA	Titratable acid content
SSC	Soluble solids content
VC	Vitamin C content
LYC	Lycopene content
R^2^	The coefficient of determination
MAE	Mean absolute error
RMSE	Root mean square error
